# Beyond Glycemic Control: Exploring the Overlooked Interplay between Bladder Dysfunction, Sleep Quality, and Life Quality in Type 2 Diabetes Mellitus

**DOI:** 10.31662/jmaj.2025-0490

**Published:** 2026-02-06

**Authors:** Pranshu Chawla, Umair Mushtaq, Muhammad Sajawal Saleem, Mohammed Shafiulla Shaik, Fiza Khalid Malik, Shivam Singla, Bhavna Singla, Haniyya Hussain C.P., Sunita Kumawat, Asreena Arayilakath Pattuvathil, Sana Saleem Tariq

**Affiliations:** 1 Department of Emergency Medicine, Dashmesh Hospital, New Delhi, India; 2 Department of Psychiatry, Birmingham and Solihull Mental Health Foundation Trust, Birmingham, United Kingdom; 3 Department of Internal Medicine, Indus Hospital & Health Network, Karachi, Pakistan; 4 Department of General Practice, Primary Medical Center PSS, Riyadh, Saudi Arabia; 5 Department of General Practice / Pediatrics, University of Health Sciences, Lahore, Pakistan; 6 Department of Internal Medicine, Tidal Health Peninsula Regional, Salisbury, MD, USA; 7 Department of Internal Medicine, Erie County Medical Center, Buffalo, NY, USA; 8 Department of Medicine, Burjeel Holdings, Abu Dhabi, United Arab Emirates; 9 Department of Internal Medicine, PWC / St. Francis Medical Center, Lynwood, CA, USA; 10 Department of Anesthesia, Hamad Medical Corporation, Doha, Qatar; 11 Department of Internal Medicine, Islamabad Medical and Dental College, Islamabad, Pakistan

**Keywords:** type 2 diabetes mellitus, bladder dysfunction, sleep quality, quality of life

## Abstract

**Introduction:**

Type 2 diabetes mellitus (T2DM) is often comorbid and, along with non-glycemic complications such as bladder dysfunction and sleep disturbances, significantly reduces the health-related quality of life (HRQoL). In Pakistan, these associations have remained unexplored. The present study sought to investigate whether diabetic bladder dysfunction (DBD) was associated with the sleep quality and HRQoL in adults with T2DM.

**Methods:**

This study employed a cross-sectional design among 300 T2DM patients selected from outpatient departments in Islamabad and Rawalpindi using convenience sampling. The instruments were validated using the International Consultation on Incontinence Questionnaire-Short Form, Pittsburgh Sleep Quality Index, and Short Form-36 Health Survey. Pearson correlations, t-tests, analysis of variance, and multiple linear regression were used to analyze the data.

**Results:**

Poor sleep quality was positively associated with urinary incontinence (r = 0.472, p < 0.001) and negatively correlated with HRQoL (r = -0.487 to -0.561, p < 0.001). Urinary incontinence was also negatively associated with HRQoL (r = -0.446 to -0.528, p < 0.001). Regression analyses showed that poor sleep (β = -0.289 to -0.312), urinary incontinence (β = -0.218 to -0.228), age (β = -0.164 to -0.176), and female gender (β = -0.152 to -0.162) were independently associated with lower HRQoL (p < 0.01).

**Conclusions:**

Poor sleep and bladder dysfunction are independent and collective contributors to poor quality of life in T2DM. The inclusion of these factors, along with glycemic control, could lead to more comprehensive diabetes care.

## Introduction

Type 2 diabetes mellitus (T2DM) is a persistent metabolic condition whose prevalence is growing at an alarming rate globally, especially in low-income countries. It arises from defective insulin secretion and action, as well as genetic and environmental factors that contribute to disturbed glucose homeostasis and increased cardiovascular disease risk ^(1), (2)^. Although glycemic targets provide the basis for T2DM management, disease progression and its complications are highly variable ^(3)^.

Diabetic bladder dysfunction (DBD), also known as lower urinary tract symptoms, is among the most prevalent and underdiagnosed comorbidities in diabetes ^(4)^. It affects almost one-half of all patients with diabetes; however, it is underdiagnosed. DBD involves impaired sensation of the bladder, incomplete emptying, detrusor overactivity, and storage symptoms, which significantly affect quality of life (QoL) ^(5), (6)^.

Preliminary evidence indicates sleep quality is a mediator of these associations. Lower urinary tract symptoms in diabetics are linked to poor sleep quality, which exacerbates health-related QoL (HRQoL) ^(7)^. Poor sleep quality and diminished sleep efficiency are also connected with inadequate glycemic control and an increased likelihood of microvascular complications ^(8)^. Additionally, poor sleep itself is associated with diabetes-specific QoL, and autonomic dysfunction has been associated with nocturnal micturition ^(9), (10)^.

T2DM is increasingly prevalent in Pakistan. In primary healthcare, management primarily focuses on glycemic control, often overlooking complications such as urinary incontinence (UI), as a manifestation of DBD and sleep disturbances, which can affect productivity, psychosocial functioning, and QoL. Cultural barriers, limited clinician awareness, lack of local evidence, and stigma compound this neglect. This study aims to provide region-specific data on the demographic and clinical characteristics of adults with T2DM, to describe the occurrence and patterns of UI as a manifestation of DBD, and to examine the associations among sleep quality, bladder dysfunction, and health-related QoL in this population.

## Materials and Methods

A cross-sectional design was used in the present study to examine the relationships among UI, as a manifestation of DBD, sleep quality, and general QoL in Pakistani adults with T2DM. The outpatient departments of the participating hospitals in Islamabad and Rawalpindi were selected for participant recruitment. Demographic data, diabetes history, urinary symptoms, sleep quality, and overall QoL information were obtained with a structured questionnaire.

Qualified research assistants approached patients, introduced the study’s purpose, and answered any questions participants had before obtaining informed consent. The participants were assured of confidentiality and were informed that they had the right to withdraw at any time without facing any negative consequences. This approach enabled culturally appropriate communication, reduced potential participant discomfort, and promoted the collection of sound, relevant data within the local Pakistani context.

### Recruitment and sample

The prevalence (p) was assumed to be 0.5, and the population with T2DM (p) was considered to be infinite, as no local data on the pooled prevalence of UI, as a manifestation of DBD, and sleep disturbances among Pakistani adult patients with T2DM, were available. A 95% confidence level (Z = 1.96) and a margin of error of 0.05 were used to estimate the sample size ^(11)^. Participants were recruited from the outpatient departments of some selected hospitals in Islamabad and Rawalpindi using a convenience sampling technique.

Of 350 individuals approached, 50 were either ineligible or refused to participate. The remaining 300 respondents provided informed consent, completed the survey questionnaire, and were incorporated in the final analysis.

### Eligibility criteria

Patients aged 18 years and above with diagnosed T2DM were included in the study during outpatient clinics at the respective hospital wards in Islamabad and Rawalpindi. All participants were required to be able to read and answer the study questionnaire and provide informed consent. Patients with neurological diseases, urinary tract infections, a history of urological surgery, psychological disorders, or other conditions that might independently affect bladder function or sleep were excluded. Pregnant women and individuals with type 1 diabetes mellitus were also excluded to maintain a homogeneous study population.

### Instruments and procedures

A structured questionnaire was designed for this survey, comprising two sections: demographic and clinical characteristics of the participants, as well as standardized questionnaires on bladder function, sleep quality, and QoL. All tools were administered in English, with a standardized verbal explanation in Urdu provided to participants by trained research assistants to ensure comprehension and consistency.

### Demographic information

The first part of the questionnaire included demographic and clinical data related to T2DM. This included age, sex, marital status, educational background, occupational background, diabetes duration, treatment type, and the presence of other medical conditions (e.g., hypertension, heart disease, kidney disease, or others), which were recorded to account for potential confounding effects on sleep quality, UI, and HRQoL. These variables provided the groundwork for subgroup analysis, placing relationships among demographic factors, bladder dysfunction, sleep quality, and QoL in context.

### Pittsburgh sleep quality index

Sleep quality was measured using the Pittsburgh sleep quality index (PSQI), a self-administered questionnaire with 19 items divided into seven components: subjective sleep quality, sleep latency, sleep duration, habitual sleep efficiency, sleep disturbances, use of sleep medication, and daytime dysfunction. The global PSQI score was calculated by summing the seven component scores, following the original scoring method proposed by Buysse et al. ^(12)^. Each item is scored 0-3. Component scores were summed to produce a global PSQI score ranging from 0 to 21, with higher scores indicating poorer sleep quality. The PSQI has been shown to have good reliability, with a reported Cronbach’s alpha of 0.83 ^(12)^.

### International consultation on incontinence questionnaire

Bladder function was assessed using the International Consultation on Incontinence Questionnaire-Short Form (ICIQ-SF). This questionnaire was developed by Avery et al. ^(13)^ in 2004 as a reliable and straightforward screening tool for UI in adults. The survey typically takes 5-7 minutes to complete. This summation approach follows the original scoring procedure and has been widely used in prior studies as a valid measure of overall incontinence severity. It consists of four main components. The first three items are then totaled to produce a maximum score between 0 and 21, with higher scores indicating increasing severity of incontinence. The ICIQ-SF has also demonstrated good internal consistency (Cronbach’s alpha = 0.88) ^(13)^.

### Short Form-36 Health Survey

In this study, the Short Form-36 Health Survey (SF-36) was used to assess HRQol. Initially developed by Ware and Sherbourne in 1992, the SF-36 evaluates eight health domains. The Physical Component Summary (PCS) and Mental Component Summary (MCS) scores were calculated according to the standard scoring algorithm, with higher scores indicating better perceived health. Scores for each domain range from 0 through 100, with higher scores representing better perceived health in that domain. The SF-36 has demonstrated high reliability across various populations with chronic diseases, with a Cronbach’s alpha coefficient of 0.90, showing strong internal consistency. Permission to use the original version of the questionnaire was obtained from the RAND Corporation, and due credit has been given for its creation ^(14), (15)^.

### Procedure and ethical considerations

The study was conducted following approval by the institutional review board (086/IMDC/IRB/24) of Islamabad Medical and Dental College. Data collection was carried out from June 2025 to September 2025. The study aims were provided, and written informed consent was obtained, assuring confidentiality, voluntary participation, and the freedom to withdraw at any time. A structured, self-administered questionnaire containing demographic and clinical data was administered, and trained research assistants were available to provide support, ensuring completion with minimal discomfort. Returned questionnaires were inspected for completeness, coded, and safely stored away for analysis. All procedures adhered to the ethical principles described in the Declaration of Helsinki, ensuring the privacy, confidentiality, and cultural respect of the subjects.

### Analysis

Data was analyzed using IBM SPSS Statistics 26. Demographic and clinical variables were summarized using descriptive statistics, such as frequencies and percentages. For PSQI and ICIQ-SF, missing items within a domain were imputed using the mean of completed items if ≤1 item was missing; otherwise, the domain score was excluded. SF-36 missing items were handled according to the standard SF-36 scoring protocol. Pearson’s correlation, independent-samples t-tests, and one-way analysis of variance (ANOVA) with effect sizes expressed as partial eta-squared and Cohen’s d were employed to assess the associations among sleep quality, UI, and HRQoL. Factors associated with HRQoL were identified using multiple linear regression, and unstandardized and standardized coefficients, standard errors, t-values, *p*-values, and 95% confidence intervals were reported. A histogram plot was used to display regression coefficients. Statistical significance was defined as 0.05.

## Results

### Baseline demographic data of participants

The demographic profile of 300 participants is given in Table 1. The predominant age category was 60-69 years (N = 70, 23%), with 120 (40%) men and 180 (60%) women. The majority were married (N = 117, 39%) or single (N = 110, 37%). The education level ranged from primary (N = 94, 31%) to graduate/postgraduate (N = 15, 5%). Occupation consisted of employed (N = 109, 36%) and housewives (N = 104, 35%). The duration of diabetes was 6-10 years in 102 (34%) participants; treatment included insulin therapy (N = 112, 37%) and diet/lifestyle changes (N = 50, 17%). Hypertension (N = 105, 35%) was as frequent as previous smoking (N = 139, 46%).

**Table 1.** Participant Demographics (N = 300).

Variable	f (N)	%
Age	-	-
30-39 years	60	20
40-49 years	50	17
50-59 years	65	22
60-69 years	70	23
69 years and above	55	18
Gender	-	-
Male	120	40
Female	180	60
Marital status	-	-
Single	110	37
Married	117	39
Widowed	61	20
Divorced/separation	12	4
Educational level	-	-
No formal education	61	20
Primary	94	31
Secondary	83	28
Higher secondary	47	16
Graduate/postgraduate	15	5
Occupation	-	-
Unemployed	45	15
Employed	109	36
Housewife	104	35
Retired	42	14
Duration of type 2 diabetes	-	-
Less than 1 year	50	17
1-5 years	91	30
6-10 years	102	34
More than 10 years	57	19
Current treatment for diabetes	-	-
Diet and lifestyle modification only	50	17
Oral hypoglycemic agents	87	29
Insulin therapy	112	37
Both oral agents and insulin	51	17
Presence of Other Medical Conditions	-	-
None	57	19
Hypertension	105	35
Heart disease	86	29
Kidney disease	34	11
Others	18	6
Smoking status	-	-
Never smoked	78	26
Former smoker	139	46
Current smoker	83	28

Note. f=frequency, %=percentage; Values are presented as N (%), N = 300; No statistical comparisons were performed for demographic variables in this table

### Correlation analysis

Table 2 demonstrates that poor sleep quality (greater PSQI scores) was significantly related to more UI (r = 0.472, p < 0.001), less physical (r = -0.561, p < 0.001), and mental QoL(r = -0.487, p < 0.001). Equally, the physical (r = -0.528, p < 0.001) and mental (r = -0.446, p < 0.001) quality-of-life scores were negatively correlated with UI. There was a strong positive correlation between the physical and mental components (r = 0.623, p < 0.001), indicating that they are closely associated.

**Table 2.** Pearson Correlations among sleep quality, urinary incontinence, and Quality-of-Life Components (N = 300).

Variables	1	2	3	4
Total PSQI	-	r = 0.472, t(298)=9.23, p < 0.001^**^	r= -0.561, t(298)= -11.66, p < 0.001^**^	r= -0.487, t(298)= -9.61, p < 0.001^**^
Total ICIQ-UI SF	-	-	r= -0.528, t(298)= -10.71, p < 0.001^**^	r= -0.446, t(298)= -8.58, p < 0.001^**^
PCS	-	-	-	r = 0.623, t(298)= 13.72, p < 0.001^**^
MCS	-	-	-	-

Values represent Pearson correlation coefficients (r) between continuous variables; N = 300; p < 0.001 (2-tailed) was considered statistically significant and is denoted with double asterisks (**). ICIQ-UI SF: urinary incontinence short form; MCS: mental component summary; PCS: physical component summary; PSQI: Pittsburgh sleep quality index.

### Gender differences

Table 3 indicates a significant gender difference across all variables in the study. Women had worse sleep quality (PSQI: 9.15 ± 2.34) and a higher level of urinary incontinence severity (ICIQ-UI SF: 14.18 ± 3.16) than males (6.84 ± 2.18 and 11.42 ± 3.02, respectively; p < 0.001). On the other hand, males had higher scores on physical (PCS: 52.46 ± 8.12) and mental (MCS: 50.21 ± 7.60) quality-of-life aspects than females (47.38 ± 7.85 and 45.59 ± 7.91, respectively; p < 0.001). All differences were significant, with large effect sizes, indicating that females reported worse sleep, more bladder dysfunction, and poorer overall QoL.

**Table 3.** Comparison of Sleep Quality, Bladder Dysfunction, and Quality-of-Life Components between Male and Female Participants (N = 300).

Variable	Male (N = 120; 40%) M ± SD	Female (N = 180; 60%) M ± SD	*t*	*p*	95% CI LL	95% CI UL	Cohen’s *d*
Total PSQI	6.84 ± 2.18	9.15 ± 2.34	-8.27	<0.001^**^	-2.84	-1.79	0.92
Total ICIQ-UI SF	11.42 ± 3.02	14.18 ± 3.16	-7.24	<0.001^**^	-3.53	-1.99	0.84
PCS	52.46 ± 8.12	47.38 ± 7.85	5.28	<0.001^**^	3.16	7.00	0.61
MCS	50.21 ± 7.60	45.59 ± 7.91	4.94	<0.001^**^	2.71	6.53	0.57

Values are presented as mean ± standard deviation (M ± SD); Independent samples t-tests were conducted to compare participants of both male and female groups; Group sizes are shown as N (%); Reported statistics include p-values, t-values, 95% confidence intervals (CI), and effect sizes (Cohen’s d); A p < 0.001 was considered statistically significant, N = 300. ICIQ-UI SF: urinary incontinence short form; MCS: mental component summary; PCS: physical component summary; PSQI: Pittsburgh sleep quality index.

### Age-group comparisons

Table 4 shows considerable age variation across all variables in the study. The older participants reported worse sleep quality and increased bladder dysfunction, as indicated by higher PSQI and ICIQ-UI SF scores (p < 0.001). Mean PSQI scores increased gradually with age between the 30-39 and 69 years and above age groups, with the age-group score rising from 6.42 to 10.36, and the UI score rising from 10.38 to 15.12. Conversely, both the PCS and MCS quality-of-life scores declined markedly with age, suggesting lower well-being among older participants. All ANOVA comparisons were significant (p < 0.001) with moderate effect sizes (η 2 = 0.100-0.162), indicating that older age was strongly correlated with poor sleep, greater bladder dysfunction, and lower QoL.

**Table 4.** Comparison of Sleep Quality, Bladder Dysfunction, and Quality-of-Life Components across Age Groups (N = 300).

Variable	30-39 years (N = 60; 20%) M ± SD	40-49 years (N = 50; 17%) M ± SD	50-59 years (N = 65; 22%) M ± SD	60-69 years (N = 70; 23%) M ± SD	≥69 years (N = 55; 18%) M ± SD	*F*(4,295)	*p*	η^2^
Total PSQI	6.42 ± 2.15	7.08 ± 2.31	8.21 ± 2.42	9.84 ± 2.51	10.36 ± 2.70	14.26	<0.001^**^	0.162
Total ICIQ-UI SF	10.38 ± 2.85	11.25 ± 3.01	13.48 ± 3.24	14.76 ± 3.10	15.12 ± 3.28	11.17	<0.001^**^	0.132
PCS	53.85 ± 7.65	51.74 ± 8.02	48.32 ± 7.84	45.78 ± 7.62	43.95 ± 8.04	9.38	<0.001^**^	0.113
MCS	51.12 ± 7.58	49.83 ± 7.70	46.92 ± 7.80	44.36 ± 7.66	42.15 ± 7.94	8.22	<0.001^**^	0.100

Data are presented as mean ± standard deviation (M ± SD); Group sizes are shown as N (%); One-way ANOVA was conducted to examine the effect; All comparisons were significant at p <0.01, <0.001; η^2^ represents partial eta-squared effect size. ANOVA: analysis of variance; ICIQ-UI SF: urinary incontinence short form; MCS: mental component summary; PCS: physical component summary; PSQI: Pittsburgh sleep quality index.

### Factors associated with QoL

Table 5 presents the multiple regression analysis of factors associated with PCS and mental MCS quality-of-life scores. Lower PCS (-0.289) and MCS (-0.312) scores were significantly associated with poor sleep quality (higher PSQI) and greater severity of urinary incontinence (higher ICIQ-UI SF). Other negative factors associated with both PCS and MCS were also significant (increasing age, female gender, and longer duration of diabetes), and other medical conditions (all p < 0.01). Conversely, current diabetes treatment was positively associated with improved physical (β = 0.132, p = 0.005) and mental (β = 0.121, p = 0.003) QoL. These findings indicate that lower sleep quality, worse incontinence, older age, female sex, and comorbidity are risk factors for poor physical and mental health, whereas active diabetes control is associated with better QoL.

**Table 5.** Multiple Regression Analysis of Factors Associated with Physical and Mental Component Summary (PCS and MCS) (N = 300).

Factors	B	SE	β	*t*	*p*	95% CI LL	95% CI UL
**Constant (PCS)**	61.482	2.105	―	29.20	<0.001^***^	57.34	65.62
Total PSQI	-0.482	0.091	-0.289	-5.29	<0.001^***^	-0.662	-0.302
Total ICIQ-UI SF	-0.356	0.084	-0.218	-4.24	<0.001^***^	-0.522	-0.190
Age	-0.182	0.052	-0.164	-3.50	<0.001^***^	-0.284	-0.080
Gender (1 = male, 2 = female)	-1.846	0.542	-0.152	-3.41	0.001^**^	-2.913	-0.779
Duration of type 2 diabetes	-0.315	0.107	-0.143	-2.94	0.004^**^	-0.525	-0.105
Current treatment for diabetes	0.824	0.287	0.132	2.87	0.005^**^	0.258	1.390
Presence of other medical conditions	-0.642	0.213	-0.148	-3.01	0.003^**^	-1.063	-0.221
**Constant (MCS)**	58.724	2.386	―	24.61	<0.001^***^	54.02	63.43
Total PSQI	-0.534	0.102	-0.312	-5.24	<0.001^***^	-0.735	-0.333
Total ICIQ-UI SF	-0.382	0.095	-0.228	-4.02	<0.001^***^	-0.570	-0.194
Age	-0.204	0.058	-0.176	-3.52	<0.001^***^	-0.318	-0.090
Gender (1 = male, 2 = female)	-2.156	0.611	-0.162	-3.53	<0.001^***^	-3.358	-0.954
Duration of type 2 diabetes	-0.278	0.092	-0.131	-3.02	0.003^**^	-0.459	-0.097
Current treatment for diabetes	0.752	0.248	0.121	3.03	0.003^**^	0.264	1.240
Presence of other medical conditions	-0.695	0.225	-0.142	-3.09	0.002^**^	-1.139	-0.251

Multiple linear regression was conducted to identify factors associated with Physical and Mental Component Summary (PCS and MCS) scores; Values include unstandardized coefficients (B), 95% confidence intervals (CIs), standard error (SE), standardized beta coefficients (β), and *p*-values; **p < 0.01, ***p < 0.001 was considered statistically significant, N = 300. ICIQ-UI SF: urinary incontinence short form; PSQI: Pittsburgh sleep quality index.

### Factors associated with QoL in T2DM

Standardized beta (β) coefficients from a regression analysis examining associations with HRQoL (SF-36) in a sample of 300 participants are shown in Figure 1. PSQI had the strongest negative association (β = -0.331), indicating that inadequate sleep is significantly linked with lower QoL. Other factors, including UI as a manifestation of DBD (ICIQ-UI SF), age, duration of diabetes, and gender, were also negatively associated with HRQoL to varying degrees. Current diabetes treatment was positively associated with QoL (β = 0.107), suggesting a modest relationship.

**Figure 1.**
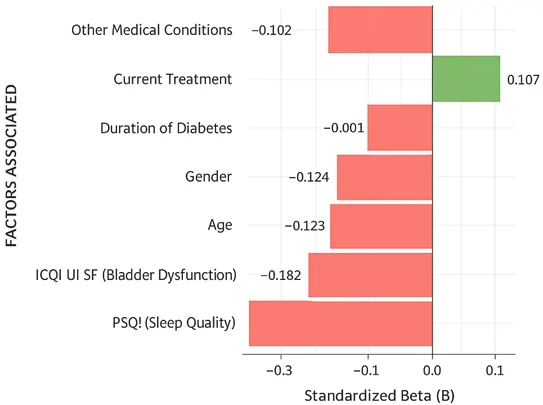
Standardized regression coefficients for factors associated with physical and mental QoL (PCS and MCS) (N = 300). MCS: mental component summary; PCS: physical component summary; QoL: quality of life.

## Discussion

This study demonstrates that UI, as a manifestation of DBD, and poor sleep quality are significant, independent factors associated with reduced HRQoL in adults with T2DM. In our study, poor sleep quality was moderately correlated with higher UI scores. This finding is consistent with a study reporting poorer sleep quality among individuals with UI and more frequent nocturia ^(16)^. Lower SF-36 scores were also strongly associated with sleep disturbances, underscoring the importance of sleep for total HRQoL ^(17)^. The results of our study suggested a moderate to strong relationship between lower HRQoL and higher UI. This correlates with a systematic review and meta-analysis indicating that people with UI score considerably lower on the SF-36 than those without incontinence ^(18)^.

Women have a lower quality of sleep and more severe urinary incontinence than men, which is consistent with population-based studies that have established that women are more susceptible to poor sleep and UI ^(19), (20)^. Correspondingly, Males had higher scores on both the PCS and MCS components than females, despite having similar sociodemographic factors ^(21)^.

In our study, sleep quality was negatively associated with increasing age. This finding aligns with previous research, which also reports that older adults tend to have lower PSQI scores, indicating poorer sleep quality ^(22)^. Age is a significant factor in the severity of UI in our study. This finding aligns with past studies, which reported a high prevalence of UI in older women, with age identified as a significant risk factor, highlighting the cumulative association of ageing on bladder function ^(23)^. In our research, health-related QoL decreased significantly with increasing age. This finding aligns with prior studies on healthy adults, which have also reported lower SF-36 scores among older age brackets, indicating a small yet consistent influence of aging on HRQoL ^(24)^.

The multiple regression analysis revealed that sleep quality was the strongest independent factor associated with HRQoL, followed by UI and age, with female gender playing a significant role, albeit with a lesser contribution ^(17), (18), (21), (24)^. The results showed that the longer the duration of diabetes, the lower the HRQoL, which is in line with other studies that have reported a slight but significant decrease in SF-36 scores with each year of living with T2DM ^(25)^. According to our findings, current treatment is positively correlated with HRQoL, as earlier studies have reported that active management of diabetes enhances patients’ perceived QoL ^(26)^. We found that the presence of other medical conditions was associated with lower HRQoL, consistent with previous studies showing that comorbidities, including arthritis and urinary diseases, are linked with reduced QoL ^(27)^.

The results underscore the importance of multifactorial determinants of HRQoL in adults with T2DM, highlighting the need to implement integrated interventions that target sleep quality, bladder function, disease management, and control of comorbidities to enhance QoL for patients.

### Limitations and future directions

This research has some limitations that must be noted. Its cross-sectional nature prevents causal interpretation of the relationships among UI as a manifestation of DBD, sleep quality, and HRQoL; longitudinal studies are required to determine these relationships over time and the effects of interventions. The use of English-language questionnaires may have introduced measurement bias, as participants’ language proficiency was not formally assessed. Future studies should consider using culturally adapted and validated Urdu versions to improve response accuracy. In addition, other potential confounders, such as body mass index, educational level, depressive symptoms, medication use, and lifestyle factors, were not accounted for, potentially leading to residual confounding. Convenience sampling was used in Islamabad and Rawalpindi hospitals, which may limit the generalizability of the study to the broader Pakistani population. Additionally, the use of self-administered questionnaires can introduce recall and reporting bias. Furthermore, biochemical markers, such as glycated hemoglobin A1c or neuropathy indicators, are not utilized, which limits physiological correlations. Future studies should utilize prospective study designs that include biological, psychosocial, and lifestyle factors, and consider specific ways to improve HRQoL, such as sleep hygiene education, pelvic floor rehabilitation, and optimized glycemic control. Creating culturally modified urinary and sleep disturbance screening instruments can further enhance early diagnosis and patient-centered diabetes care.

### Conclusions

The study emphasizes that inadequate sleep quality and bladder dysfunction are two common and interdependent problems that significantly lower the HRQoL in patients with T2DM. These correlations remain significant despite controlling for demographic and clinical variables, indicating that both sleep and urinary health require a higher clinical priority in the care of diabetes. Discussing these areas of frequent neglect can help build a more comprehensive patient-centered model of care that focuses on improving overall health rather than solely on glycemic control.

## Article Information

### Author Contributions

Conceptualization, study design, and supervision: Pranshu Chawla.

Data collection, clinical input, and methodology: Umair Mushtaq.

Statistical analysis, data validation, and results interpretation: Muhammad Sajawal Saleem.

Patient recruitment and clinical validation of findings: Mohammed Shafiulla Shaik.

Literature review and drafting of the introduction and discussion: Fiza Khalid Malik.

Data curation and manuscript writing (methods and results sections): Shivam Singla.

Editing, critical revision, and quality assurance of the manuscript: Bhavna Singla.

Data acquisition and contribution to the discussion section.: Haniyya Hussain C.P.

Literature review, referencing, and formatting: Sunita Kumawat.

Methodological guidance and review of clinical implications: Asreena Arayilakath Pattuvathil.

Corresponding author: project administration, manuscript coordination, and final approval of the version to be published: Sana Saleem Tariq.

All authors meet the ICMJE criteria for authorship, approved the final version of the manuscript, and agree to be accountable for all aspects of the work.

### Conflicts of Interest

None

### IRB Approval Code and Institution

Ethical approval was obtained from the Institutional Review Board of Islamabad Medical & Dental College (IRB Approval Code: 086/IMDC/IRB/24).
